# Effects of communication delay in the dual cockpit remote robotic surgery system

**DOI:** 10.1007/s00595-023-02784-9

**Published:** 2023-12-09

**Authors:** Yoshiya Takahashi, Kenichi Hakamada, Hajime Morohashi, Yusuke Wakasa, Hiroaki Fujita, Yuma Ebihara, Eiji Oki, Satoshi Hirano, Masaki Mori

**Affiliations:** 1 Department of Gastroenterological Surgery, Hirosaki University Graduate School of Medicine, 5 Zaifu-cho Hirosaki, Aomori, Tokyo, 036-8562 Japan; 2 Committee for Promotion of Remote Surgery Implementation, Japan Surgical Society, Tokyo, Japan; 3 Faculty of Medicine, Department of Gastroenterological Surgery II, Hokkaido University, Sapporo, Japan; 4 Department of Surgery and Science, Kyushu University, Fukuoka, Japan; 5 Tokai University School of Medicine, Isehara, Japan

**Keywords:** Robotic surgery, Telesurgery, Dual console

## Abstract

**Purpose:**

To evaluate the impact of dual cockpit telesurgery on proctors and operators, and acceptable levels of processing delay for video compression and restoration.

**Methods:**

Eight medical advisors and eight trainee surgeons, one highly skilled per group, performed gastrectomy, rectal resection, cholecystectomy, and bleeding tasks on pigs. Using the Medicaroid surgical robot hinotori^™^, simulated delay times (0 ms, 50 ms, 100 ms, 150 ms, and 200 ms) were inserted mid-surgery to evaluate the tolerance level. Operative times and dual cockpit switching times were measured subjectively using 5-point scale questionnaires (mSUS [modified System Usability Scale], and Robot Usability Score).

**Results:**

No significant difference was observed in operative times between proctors and operators (proctor: *p* = 0.247, operator: *p* = 0.608) nor in switching times to the dual cockpit mode (*p* = 0.248). For each survey setting, proctors tended to give lower ratings to delays of ≥ 150 ms. No marked difference was observed in the operator evaluations. On the postoperative questionnaires, there were no marked differences in the mSUS or Robot Usability Score between the proctors and operators (mSUS: *p* = 0.779, Robot Usability Score: *p* = 0.261).

**Conclusion:**

Telesurgery using a dual cockpit with hinotori^™^ is practical and has little impact on surgical procedures.

## Introduction

Robotic surgery has spread to facilities in various parts of Japan since the 2018 revision of medical reimbursement regulations began allowing insurance coverage of robotic surgery in various fields, including gastrointestinal and respiratory surgery. In addition, new surgical robots are entering the market, and new technologies are being developed [1]. One of the advantages of robotic surgery is that it enables us to make various innovations based on digitized information. Surgical education methods using swapping and annotation functions with a dual console go beyond what was possible with conventional approaches and are quickly becoming standard protocols among the more useful methods of surgical education. In recent years, research has been conducted to implement these more evolved system tools in society through telesurgery technology, using robots and telecommunication modalities.

We have been conducting experimental studies on telesurgery since February 2021 and have verified the effects of communication delays and image degradation on surgical operations [2–6]. We have demonstrated the feasibility of remote surgery using Japanese commercial lines and domestic robots and developed guidelines for clinical applications. However, we have not studied the construction of a system in which a physician at an institutional hospital in a remote location can provide surgical support through a dual cockpit to a physician performing surgery locally.

One of the main problems with telesurgery systems is communication delays. To implement remote surgical support in society, it is essential to develop tools that provide guidance to local surgeons from remote locations. Therefore, it is necessary to verify the acceptable delay in the surgical support system.

The present study evaluated the acceptable range of communication delays for teaching using a dual cockpit in telesurgery.

## Methods

### Operation robot

A hinotori^™^ surgical support robot was used in this study. This technology, developed by Medicaloid, consists of three units: the Operation Unit (OU), surgeon’s cockpit (SC), and Vision Unit (VU). The operator and proctor performed the surgery by operating the hand controller while viewing the three-dimensional monitor in the SC.

### Communication environment

**Fig. 1:**
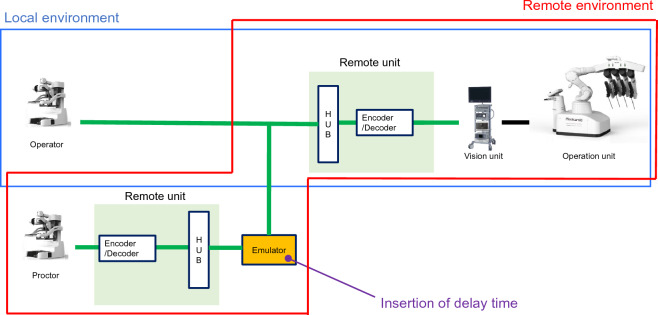
The operation unit (OU), surgeon cockpit (SC), and vision unit (VU) used by the surgeon were installed in the same room, making it a local environment. The SC for the proctor was installed in a separate room to create a pseudo-remote environment

The surgeons’ SC, OU, and VU were set up in the same room. The proctor's SC and VU were set up in an adjacent room and connected via an encoder/decoder and emulator to create pseudo-telesurgery conditions (Fig. 1). The emulator was then used to insert delay times. Conditions of 0, 50, 100, 150, and 200 ms were set for gastrectomy, rectal resection, and cholecystectomy and 150 and 200 ms for the bleeding task.

### Robot tasks

Eight surgeons (proctors; specialists in the Japan Robotic Surgery Society and equivalent qualifications) and eight trainee surgeons (operators; no experience performing robotic surgery or currently seeking to qualify for robotic surgery) were used as subjects and were divided into eight groups of two, with each consisting of one proctor and one operator. The experiment was conducted over two days, with one pig operated on each day. Four surgical teams participated per day and were asked to perform each organ task, resulting in a total of eight teams for validation. After practicing with hinotori^™^ in a separate room, the subjects moved on to the evaluation task. Each task was performed in the following order: 0, 50, 100, 150, and 200 ms. The subjects were asked to operate each setting for approximately 15 min before moving on to the next setting in succession. The task content was set in advance with instructional points (described below), and the proctor provided instructional intervention under pseudo-remote conditions based on these points.

### Instructional points

- Gastrectomy: Gastroepiploic arteriovenous dissection to duodenectomy, opening of the lesser omentum, ligation and dissection of the right gastric artery, and suprapancreatic margin dissection.
- Rectal resection: medial approach to inferior mesenteric artery, total mesorectal excision
- Cholecystectomy: Calot triangle processing, liver bed dissection
- Bleeding task: the operator intentionally injures the mesenteric artery or arteriovenous vein running through the broad ligament of the uterus, and the proctor performs suture hemostatic manipulation.

### Animals

Two pigs, weighing 36.24 and 38.82 kg, were used. Both were sedated with intramuscular injections of ketamine (10 mg/kg) and xylazine (2 mg/kg), and muscle relaxation was performed using 0.6 mg/kg of rocuronium. After completion of the experiment, cardiac arrest was induced by deep anesthesia, de-bleeding, and intravenous infusion of KCl-saturated water to euthanize the animals (Approval Nos. 087 and 088, respectively).

### Evaluation

The following five-point rating scale was used: 1, Totally impossible; 2, Partially possible but almost impossible; 3, Uncomfortable but possible; 4: Some discomfort but possible; 5, Possible. The subjects were asked to respond subjectively after completing the task in each setting. A higher number indicates a higher evaluation.

A comment space was also provided for the respondents to freely describe how they felt during the task.

### Modified version of the system usability scale created by brook (mSUS)

The usefulness of telesurgery was evaluated using the mSUS [7]. Each of the nine items was rated on a five-point scale, and the total score was tabulated. The total possible score was 45, with higher scores indicating greater usefulness of the telesurgery system. The subjects were asked to answer questions after each organ task was completed.

### Robot usability score

To evaluate the operability of the surgical robot, an evaluation table was created by modifying its usability score [8]. Each of the eight items was rated on a five-point scale, and the total score was tabulated. The maximum score was 40, with higher scores indicating better operability of the surgical robot. Responses were obtained from the subjects after completion of each organ task.

### Statistical analyses

The EZR software program (Jichi Medical University) was used [9]. Tests of normality were performed using the Kolmogorov–Smirnov test, and tests of equal variance were performed using the Bartlett test; the analysis was performed using the Mann–Whitney *U* test, and statistical significance was determined with *p* < 0.05.

## Results

### Operation time

Both the proctor and operator measured the time from the start of the robotic forceps movement to the end of the movement as the operation time. The time for each operation was measured for each switch of the operating authority and was calculated as the accumulated operation time for both the proctor and the operator at the end of one team. The mean operative time for the proctor was 216 s in the 0 ms delay setting, 245 s at 50 ms, 152 s at 100 ms, 302 s at 150 ms, and 246 s at 200 ms (*p* = 0.247). The mean operative time for the operator was 433 s in the 0-ms delay setting, 559 s at 50 ms, 610 s at 100 ms, 546 s at 150 ms, and 613 s at 200 ms (*p* = 0.608). The switching time between proctor and operator was 2.91 s at the 0 ms delay setting, 4.19 s at 50 ms, 3.46 s at 100 ms, 3.02 s at 150 ms, and 2.82 s at 200 ms, showing no marked effects of the delay (*p* = 0.248) (Table 1).

**Table 1:** Each operation time and switching time

	0 ms	50 ms	100 ms	150 ms	200 ms	*p* value
Proctor operation time [sec]	216 (43–412)	245 (51–390)	152 (39–285)	302 (44–470)	246 (82–389)	0.247
Operator operation time [sec]	433 (125–882)	559 (417–797)	610 (330–775)	546 (39–1412)	613 (12–1234)	0.608
Switching time [sec]	2.91 (0.84–5.67)	4.19 (1.95–5.83)	3.46 (2.19–4.66)	3.02 (1.24–4.76)	2.82 (0.85–8.11)	0.248

Values are shown as the average (range)

*p < 0.05

### 5-point scale for delays

**Fig. 2:**
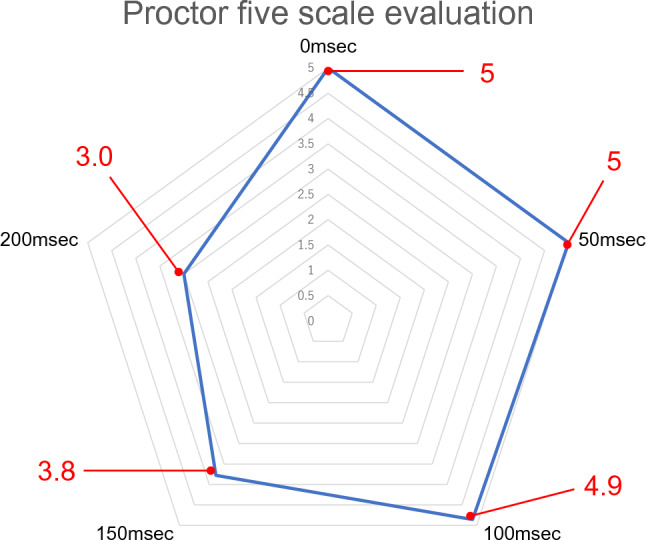
Five-point scale scores according to the proctor for each delay duration. Values represent the average

**Fig. 3:**
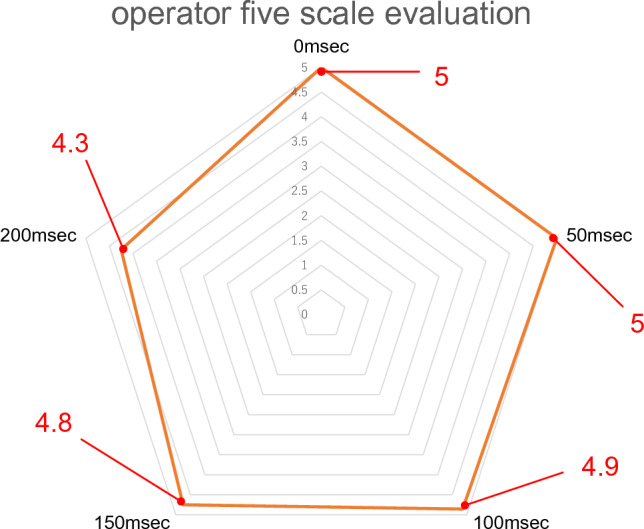
Five-point scale scores according to the operator for each delay duration. Values represent the average

The mean score on the 5-point scale evaluation by the proctors was 5 at 0 ms and 50 ms, 4.9 at 100 ms, 3.8 at 150 ms, and 3 at 200 ms, with a delay of ≥ 150 ms resulting in a low rating, indicating that instruction was difficult (*p* < 0.05). The mean 5-point scale score for the operators was 5 for delay settings of 0 and 50 ms, 4.9 for 100 ms, 4.8 for 150 ms, and 4.3 for 200 ms. The overall statistical *p*-value was 0.017, indicating that most respondents rated the instructions as viable even with a delay of ≥ 150 ms. There were no significant differences in any delay setting between groups (Figs. 2 and 3).

### Subjective evaluation questionnaires

The mSUS and robot usability scores in terms of the proctor and operator side ratings were compared. The mSUS was 37.8 for both the proctor and operator, showing no marked difference, and there was no discrepancy in the evaluation of the usefulness of the telesurgery system (*p* = 0.779). The Robot Usability Score was 29.9 for the proctor and 34.4 for the operator, indicating that the operators rated the operability of the surgical robot slightly higher than the proctors, but there was still no significant difference (*p* = 0.261) (Table 2).

**Table 2:** The mSUS and robot usability score

	Proctor	Operator	*p* value
mSUS [score]	37.8 (31–43)	37.8 (33–40)	0.779
Robot usability score [score]	29.9 (20–40)	34.4 (32–37)	0.261

Values are shown as the average (range)

*p < 0.05

## Discussion

Similar to our previous studies [10], we concluded that it is difficult for a proctor to operate in a remote environment with a delay of ≥ 150 ms. Therefore, when an unexpected situation occurs during telesurgery resulting in a delay of ≥ 150 ms, surgical support from the proctor is considered difficult. However, based on the hypothesis that the delay time limit perceived by the recipients of guidance is similar, we conducted this experiment and concluded that it is possible to receive guidance even at 200 ms, which is more than expected. This was thought to be because a certain amount of delay may be allowed for education using the annotation and swapping functions, as long as the surgery is performed smoothly.

The dual cockpit concept was introduced in the Intuitive Surgical da Vinci Si in 2009 with dual console functionality, allowing two surgeons to operate the surgical robot simultaneously [11]. The dual cockpit system is considered to be educationally useful, contributing to the improvement of surgical skills and the learning curve; this is not limited to the field of gastrointestinal surgery [12]. In addition, the dual console allows the primary surgeon and supporting physician to perform the same surgery simultaneously, making it possible to conduct surgery safely and with a high degree of reproducibility [13]. However, few reports have focused on dual cockpits in remote surgical environments. Oki et al. reported the usefulness of a dual cockpit in a remote area 140 km away, where a non-specialist surgeon performed emergency hemostasis, cholecystectomy, and renal vein ligation in surgery using pigs with remote assistance from a specialist surgeon [6].

We believe that the maximum delay time that occurs in telesurgery is limited to < 100 ms, and in our own example, the delay time was 29 ms at a distance of 150 km [3]. There are several reports of acceptable delay times, with reports of increased task times and error rates above 300 ms [14]. Similarly, surgical performance was notable worse at ≥ 300 ms than with less of a delay and showed a significantly increased error rate at ≥ 500 ms, suggesting that such delays lead to surgical risk [15]. In our report, we examined the acceptable durations of delay and found that a delay time of ≥ 100 ms affects surgical outcomes [10]. As in previous reports, we discovered that both annotation and swapping functions (≥ 150 ms) were insufficient for robotic surgical guidance from a remote location. However, it is notable that the surgeons on the receiving end of the instruction did not notice the effects of the delay as much as was felt on the instructor side. Therefore, when focusing on providing instructions from a remote location, there is a high possibility that even a delayed environment is sufficient for providing annotation and voice instruction, even if such a delay would be unacceptable when it came to operating and moving the robot.

No notable effect of delay was observed with respect to the switching time. When surgical support from a remote location is provided, it is thought that the quality of instruction is increased, provided the switching time is kept as short as possible. In addition, in an emergency situation, such as bleeding, it is desirable for the instructor to be able to instantly shift control to the proctor in order to stop any bleeding.

The significance of this experiment lies in the fact that the robot operability and environment were evaluated by not only the proctor but also the operator. The intervention of the remote instructor did not adversely affect the surgical operation of the primary surgeon, and both parties expressed their expectations concerning the usefulness of the dual cockpit remote instruction in actual clinical practice.

In this verification study, the experiments were conducted by constructing a simulated remote environment indoors, but communication modalities need to be evaluated using the commercial lines that will actually be used. As telesurgery has become more widespread, variations in the modalities used are expected to increase. There are reports that dual-console surgery using 5G and wired networks is possible [16], and it will be necessary to study and evaluate various such modalities in the future.

In the United States, the use of robots in general surgical procedures, especially for managing hernias, has become prominent, more so than in cancer surgeries, and robotic approaches are rapidly replacing most conventional approaches. In Japan, although surgical-assisting robots are becoming more widespread throughout the country, the number of surgical-assisting robots and the number of surgical slots are still insufficient to cover the total number of surgeries. Consequently, evidence of treatment outcomes is available only for a limited number of advanced centers. While robotic surgery in Japan is expected to continue to focus on cancer treatment, it is also expected to eventually follow the lead of the United States and expand its application to various general surgical procedures. With this in mind, the issue for the future is to determine how to provide surgical education to young surgeons who are just starting to use robotic surgery. There is no doubt that robotic surgery is a technology that young surgeons aim for higher quality, and more precise surgical modalities should be explored. Young surgeons working in rural areas in particular will benefit greatly from remote surgical training from a medical advisor at a core hospital in a more central location.

In this verification study, telesurgery with a dual console using hinotori^™^ was practical, and the acceptable delay between the proctor and operator was clarified. When considering the characteristics of each hospital and region, it is safe to assume that environmental conditions will vary widely, such as what kind of communication modality will be used to connect the core hospital and the remote hospital and which surgical robot will be used. Therefore, further studies under diverse conditions will be necessary in the future.

### Limitations

The experiment could not be conducted blindly because of the need to adjust the schedules of the subjects. For the same reason, a large number of subjects could not be recruited.

## Conclusion

Remote robotic surgical guidance using a dual cockpit with a hinotori^™^ device is practical. The surgeon receiving the instruction was able to follow the instruction even with a delay of ≥ 150 ms; however, the greatest acceptable communication delay for the surgeon giving the instruction was < 150 ms.
